# An improved density peaks clustering algorithm based on grid screening and mutual neighborhood degree for network anomaly detection

**DOI:** 10.1038/s41598-021-02038-z

**Published:** 2022-01-26

**Authors:** Liangchen Chen, Shu Gao, Baoxu Liu

**Affiliations:** 1 School of Computer Science and Technology, Wuhan University of Technology, Wuhan, 430063 China; 2 Institute of Information Engineering, Chinese Academy of Sciences, Beijing, 100093 China; 3 School of Applied Technology, China University of Labor Relations, Beijing, 100048 China; 4 School of Cyber Security, University of Chinese Academy of Sciences, Beijing, 100049 China

**Keywords:** Mathematics and computing, Information technology

## Abstract

With the rapid development of network technologies and the increasing amount of network abnormal traffic, network anomaly detection presents challenges. Existing supervised methods cannot detect unknown attack, and unsupervised methods have low anomaly detection accuracy. Here, we propose a clustering-based network anomaly detection model, and then a novel density peaks clustering algorithm DPC-GS-MND based on grid screening and mutual neighborhood degree for network anomaly detection. The DPC-GS-MND algorithm utilizes grid screening to effectively reduce the computational complexity, improves the clustering accuracy through mutual neighborhood degree, and also defines a cluster center decision value for automatically selecting cluster centers. We implement complete experiments on two real-world datasets KDDCup99 and CIC-IDS-2017, and the experimental results demonstrated that the proposed DPC-GS-MND can detect network anomaly traffic with higher accuracy and efficiency. Together, it has a good application prospect in the network anomaly detection system in complex network environments.

## Introduction

The rapid development of computer and communication technology has a major impact on social security and economic growth, but it is also embraced by network intruders and cyber criminals. The key targets of network attacks include smart terminals and network devices, especially smartphone providing payment function. Network security issues have become a very serious society focus^1^. It is very necessary to protect national and corporate networks, and accurately discovering abnormal is considered as an increasingly important research topic in network security. Network anomaly detection is considered an important data analysis task that can be used to identify network intrusions.

Intrusion detection approaches can be divided into misuse detection and anomaly detection. Among them, misuse detection identifies known attacks, while anomaly detection recognizes anomalies based on significant differences from normal activity. Anomaly detection is a very effective intrusion detection method, which has been applied to many application areas, such as network security system, industrial control system, and credit cards fraud detection etc.^2^. Most current network anomaly detection systems are based on supervised learning methods. However, supervised learning methods are often expensive to obtain training data, and unsupervised anomaly detection techniques can detect unknown attacks with unlabelled data. Clustering is a typical unsupervised learning method that aims to group objects into meaningful subcategories, and network traffic data can be distinguished from other data by clustering methods because they have different features by being generated from different anomaly mechanisms or normal activities^3^. However, the commonly used distance-based clustering methods cannot detect non-spherical clusters, and density-based clustering approaches may not be easy to select an appropriate threshold. This leads to low accuracy and efficiency of the existing clustering-based network anomaly detection methods.

These limitations have brought serious bottlenecks to cluster-based network anomaly detection methods. Here we propose a novel large-scale and high-dimensional network traffic anomaly detection approach, called DPC-GS-MND, which utilizes an improved density peaks clustering algorithm based on grid screening and mutual neighborhood degree. Grid screening can effectively reduce computational complexity, improve efficiency and make the algorithm independent of data size. Mutual neighborhood degree can improve the clustering accuracy. The major contributions of the paper can be summarized as follows:

1. We present a clustering-based network anomaly detection model, which includes network traffic data collection, data reduction including data sampling and dimension reduction, clustering-based anomaly detection modeling and anomaly detection results evaluation. This model can effectively handle network anomaly detection based on massive network traffic data.
2. We propose an improved density peaks clustering (DPC) algorithm called DPC-GS-MND, which combines the DPC algorithm with grid screening and mutual neighborhood degree to improve the accuracy and efficiency of network anomaly detection. In addition, we have introduced a cluster center decision value for automatically selecting cluster centers to avoid errors caused by human selection.
3. We implement complete experiments in Python and challenged it against the KDDCup99 dataset and as well as the more recent CIC-IDS-2017 one. Under the simulation condition, we show what extent our proposed DPC-GS-MND approach outperforms a basic density peaks clustering (DPC) algorithm and finally, we compare the approach to three other challengers from the literature: DPCG, MDPCA and DPC-DLP.

The rest of the paper is organized as follows. “Related works” section explains related work on network anomaly detection and DPC clustering applied in network anomaly detection. “Anomaly detection module and dataset” section introduces the whole anomaly detection module, experience dataset and pre-processing. “The DPC-GS-MND clustering algorithm” section presents the proposed DPC-GS-MND clustering algorithm and all technical points. “Evaluation” section implements complete experiments to evaluate the proposed approach on two real-world network traffic datasets. Finally, some conclusions and future research are high-lighted in “Conclusion and future work” section.

## Related works

### Network anomaly detection

**Figure 1:**
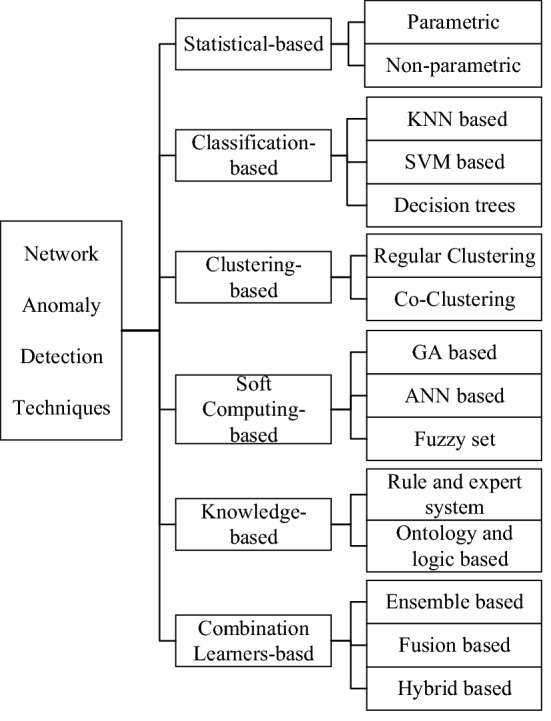
The classification of network anomaly detection techniques.

Network anomaly detection is to analyze various data collected from the network, dig complex and potential relationships, so as to infer the current network security status, and discover unforeseen attacks^4^. Researchers have applied various techniques or theories for network anomaly detection as shown in Fig. 1, such as: statistical-based, classification-based, clustering-based, soft computing, knowledge-based and combination learners-based^5^. Among them, classification-based and clustering-based methods are most broadly used in network anomaly detection systems.

Statistical-based methods apply statistical models based on network traffic distribution, and use threshold or probability conditions to identify deviating instances as anomalies. In practice, statistical-based methods have two main categories: parametric and non-parametric. Li et al.^6^ introduced statistical models based on t-distribution for network anomaly detection. Krügel et al.^7^ introduced a statistical-based processing method for detecting network anomaly or intrusion, such as R2L and U2R. HIDE^8^ is a statistical-based network anomaly detection system that applies statistical models and neural network classifiers for anomaly detection. FSAS^9^ is a statistical-based network anomaly detection system, including feature generators and flow-based detectors. Statistical-based methods can achieve high accuracy and detection rate when the threshold for identifying anomaly is correctly adjusted, and can provide accurate alarms for malicious activities without requiring prior knowledge of normal activities in advance. However, it is usually not straightforward to choose the best statistic and set the values of different parameters.

Classification-based methods rely on the normal network traffic activity profile and treats activities that deviate from the baseline profile as anomalies^10^. Several classification models have been applied to detect network anomaly, such as k nearest neighbor (KNN), support vector machines (SVM), and decision trees. The models have the ability to classify network traffic into two categories (normal or anomaly) or a set of classes (normal with each anomaly as a category)^1^. Chen et al.^11^ presented a network anomaly detection approach called FEW-NNN, which utilizes an improved KNN classification model based on fuzzy entropy weighted and natural nearest neighbor. Ambusaidi et al.^12^ proposed a least square SVM classification model to design a lightweight network anomaly detection system by selecting important network traffic features and detecting network anomalies. Abbes et al.^13^ introduced an approach that constructs an adaptive decision tree with application layer protocol analysis for effective anomaly detection. Although classification-based anomaly detection methods are popular and usually have high detection rate for known attacks, they cannot detect unknown attacks or events before providing relevant training information and require more computational resources.

Clustering-based methods cluster large datasets into similar groups without relying on class labels. The most popular types are regular clustering and co-clustering, where there are differences between the strategies for handling observations and features^10^. Specifically, regular clustering combines data points from the observations, while co-clustering considers both observations and features. Su et al.^14^ applied a network traffic sampling method based on average-linkage hierarchical clustering for network anomaly detection. Petrovic et al.^15^ proposed a network anomaly attacks method, which combines Davies-Bouldin index of the cluster and centroid diameter of the cluster. Ahmed et al.^16^ applied X-means clustering to detect collective abnormal flows. Bhuyan et al.^17^ proposed a clustering-based network anomaly detection system, which uses k-means to cluster legitimate data and computes reference points for each cluster. Clustering-based methods reduce the computational complexity and provides stable performance. However, it is difficult to evaluate the technology without assuming that the larger clusters are normal and the smaller clusters are anomalies or intrusions, and it is time-consuming to dynamically update the established configuration profiles.

Soft computing-based methods are generally thought of including genetic algorithms (GA), artificial neural networks (ANN) and fuzzy sets. Anderson et al.^18^ combined genetic algorithm and fuzzy logic to predict and detect network anomalies based on six attributes extracted from flow-based network data, in which GA algorithm is used to predict network behavior and fuzzy logic is used to evaluate whether an instance represents abnormal. Alnafessah et al.^19^ presented an anomaly detection method based on artificial neural network, which can accurately detect and classify abnormal behaviors, and can be easily used with online Spark systems. Abolhasanzadeh et al.^20^ developed a deep autoencoder technique to reduce data dimensions and then applied a shallow artificial neural networks classification model to evaluate the effectiveness. Soft computing-based methods are applied when the decision of identifying an element of network traffic as anomalous or normal is not certain. They have good efficiency and can effectively resolve inconsistency in the dataset with rough sets, but most methods have scalability problems and training becomes very difficult without a reliable amount of normal traffic data.

Knowledge-based methods construct a rule set based on the existing attack information, and then detect anomalies related to the constructed rule set. Common knowledge-based methods are rule and expert system, as well as ontology and logic-based. Snort^21^ is a popular rule-based intrusion detection system, which detects malicious network packets by matching the packets with predefined rules, and now it involves more than 20,000 rules. Petri^22^ is a knowledge-based intrusion detection system, which composes of directed bipartite graphs and colored Petri nets standing for intrusion features. Naldurg et al.^23^ proposed an IDS applying temporal logic specifications, in which attack patterns are formulated in a logic structure. Hung et al.^24^ proposed an ontology-based method to establish NADS based on the end user domain, in which a network anomaly detection system can be simply constructed. Knowledge-based methods have sufficient robustness and high accuracy to detect known attacks. However, it is impossible to identify rare or zero-day anomalies. Considering all types of anomalies or attacks, building the best, non-redundant and consistent rule set is a difficult task.

Combination learners-based methods combine different models at different levels such as features, decisions and data. They use multiple mechanisms to effectively classify data points, most of which are used for network anomaly detection systems based on ensemble-based and hybrid-based. Folino et al.^25^ proposed a distributed data mining method based on genetic programming extended with ensemble learning to improve the accuracy of anomaly detection. Perdisci et al.^26^ provided a payload network anomaly detection system based on a hybrid-based one-class SVM to improve the accuracy. Some researchers have used a combination of classifier and clustering methods to take advantage of the two technologies for network anomaly detection. Xiang et al.^27^ combined tree classifier and Bayesian clustering for network anomaly detection. Al-Yaseen et al.^28^ provided a multi-level network anomaly detection model which applies the modified k-means clustering with SVM classification and extreme learning machine. Combination learners-based methods can achieve higher accuracy and detection rates than single method, and can handle both the known and unknown attacks. However, hybridization more than one technique may lead to high computational costs and is generally not appropriate or real-time detection.

Each network anomaly detection approach can work well in certain situations. However, there is no one approach that can work well in all situations. This is because the nature of network traffic is constantly changing, and the performance of the technology depends on the point of deployment in network. Although many technologies and systems have been developed to detect network traffic anomaly, it is still necessary to develop effective technologies and systems to deal with the growing threat of cyberattacks.

### DPC algorithm in network anomaly detection

Rodriguez Alex el al. proposed density peaks clustering (DPC) algorithm in Ref^29^ in 2014. Compared with commonly used clustering algorithms, DPC algorithm is robust and efficient with only one input parameter requirements, can identify clusters of arbitrary shapes and easily find outliers. Many researchers proposed improvements to the DPC algorithm and applied them to network anomaly detection. Seyedi et al.^30^ proposed an improved DPC algorithm called DPC-DLP, which employs the idea of KNN to calculate the cut-off and local density of points, and applies a graph-based method to assign distribute points. Leung et al.^31^ provided an improved DPC with a grid-based high-dimensional clustering algorithm for anomaly detection. Xu et al.^32^ provided an improved density peaks clustering algorithm based on grid called DPCG to improve the efficiency. Ni et al.^3^ utilized unsupervised feature selection and density peaks clustering to detect network anomaly. Yang et al.^33^ provided an improved DPC algorithm called MDPCA to reduce the training scale and unbalanced samples. Li et al.^34^ proposed a hybrid model by combining KNN and DPC for network attack detection. Shi et al.^35^ presented a malicious attack detection method aiNet_DP, which combines artificial immune network and density peaks clustering.

Although DPC is a good algorithm for network anomaly detection, it still has some limitations. DPC computes local density by measuring the distance between all points, which leads to much high computational complexity, especially for large-scale data. In response to these limitations, we propose a novel improved DPC algorithm based on grid screening and mutual neighborhood degree.

## Anomaly detection module and dataset

This part focuses on a thorough description of the anomaly detection model and experience dataset, which are the most important aspects in the network anomaly detection research.

### Anomaly detection model

**Figure 2:**
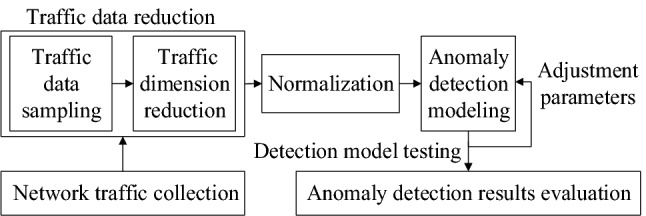
Network traffic anomaly detection design.

We design the network anomaly detection model as shown in Fig. 2. The network traffic anomaly detection process can be divided into five steps, including network traffic data collection, traffic data sampling, traffic dimension reduction, anomaly detection modeling, anomaly detection results and evaluation.

Network traffic collection: Collecting datasets that represent the problem that need to be solved is the most important step in designing a good machine learning model. The dataset employed in this research is a 10% subset of KDDCup99 dataset, which is processed by 4 GB binary TCP traffic data from 7 weeks of network. Another dataset employed in this research is CIC-IDS-2017.

Traffic data sampling: Network traffic data sampling extracts the most representative examples from the original massive network traffic dataset, removes redundant and similar traffic data and obtains a relatively small reduced traffic dataset to improve the detection performance of anomaly detection methods. Here, we sample a portion of dataset, and downsample the three kinds “neptune”, “normal” and “smurf” data to ensure relative balance with other kinds of data.

Traffic dimension reduction: Before performing anomaly detection modeling on massive high-dimensional network traffic data, it is necessary to perform feature reduction and reduction processing on the data. Here, we delete the features that can be calculated from other dimension data, and then utilize the Fisher score method and deep graph feature learning approach to obtain the key features.

Network anomaly detection modeling: After performing numerical standardization and normalization to convert all features to a common scale with zero and one, we need do network anomaly detection modeling. There are six categories anomaly detection models including statistical-based, classification-based, clustering-based, soft computing-based, knowledge-based and combination learners-based. In this paper, we implement several unsupervised clustering algorithms that do not required labelled dataset for network anomaly detection.

Anomaly detection results and evaluation: The results according to solution and clustering accuracy on the training set and the testing set will be shown in this part. Most of the network anomaly detection results use accuracy-related indicators for evaluation including false positive rate, precision rate, recall rate, overall accuracy rate and F-Value. Here, we utilize accuracy rate to evaluate the experiment results, and we compare the approach with DPC-GS-MND and three other challengers including DPCG, MDPCA and DPC-DLP.

### Dataset and preprocessing

The KDDCup99 dataset^36^ is the most commonly used dataset in the field of anomaly or intrusion detection and machine learning research. It contains 5 million connection records, which are processed by 4 GB binary data from 7 weeks of network traffic. It covers 5 main attacks classes including DoS (Denial of Service), Probe (Information gathering attacks), R2L (Root 2 Local), U2R (User 2 Root) and Normal. Each record contains 41 features, as shown in Table 1. Because of the huge amount of original data, we utilize about 494,021 records contained in 10% for experiments. In addition to about 12% of normal data, there are records of 4 traffic categories and 39 specific classes of attacks.

**Table 1:** Specific of KDD 99 10% percent.

Traffic category	Specific classes	Training size	Testing size
Normal	normal	97,278	60,593
DoS	mailbomb, smurf, teardrop, apache2, back, processtable, land, pod, neptune, udpstorm	391,458	229,853
Probe	satan, portsweep, mscan, saint, nmap, ipsweep	4107	4166
U2R	sqlattack, rootkit, xterm, perl, ps, httptunnel, buffer_overflow, loadmodule	52	228
R2L	xsnoop, xlock, ftp_write, spy, named, warezmaster, guess_passwd, phf, warezclient, worm, snmpgetattack, imap, snmpguess, multihop, sendmail	1126	16,189
Total	39 classes attacks	494,021	311,029

As shown in Table 1, the KDDCup99 10% dataset is imbalance, in which “neptune”, “normal” and “smurf” are much higher than other types, so we down-sample these three types of samples to ensure relative balance. Not all features are useful for the detection and even there will be a burden on the memory. In the pre-processing, first, we delete the features that can be calculated from others, and then, we utilize the Fisher score method and deep graph feature learning algorithm in^11^ to obtain the top 10 important features for anomaly detection, as shown in Table 2.

**Table 2:** The features used in experience.

Feature name	Feature value	Feature description
logged_in	0,1	1 if successfully logged in, else 0
dst_host_count	[0,255]	Number of connections with same dst host
count	[0,511]	Number of connections to same host as current connection
dst_host_srv_count	[0,255]	Number of connections with same dst host and service
dst_host_serror_rate	[0,1.00]	% of connections to current host with S0 errors
same_srv_rate	[0,1.00]	% of connections to the same service
dst_bytes	[0,113 billion]	Bytes from dst to src
srv_serror_rate	[0,1.00]	% of connections with same srv that have “SYN” errors
Dst_host_srv_serror_rate	[0,1.00]	% of connections to current host and specified service
serror_rate	[0,1.00]	% of connections with same dst that have “SYN” errors

Since some features are consisted of letters, such as protocol_type, flag and label, we need to convert the corresponding letters into numerical values, and then perform numerical standardization. The normalization is performed according to the follow Eq. (1) to convert the data into [0,1], where ${\mathrm{x}}_{ij}^{{\prime}}$ is the numerical standardized value of ${x}_{ij}$ and ${\mathrm{x}}_{ij}^{\prime\prime}$ is normalized value of ${\mathrm{x}}_{ij}^{{\prime}}$.

1 $$\left\{ {\begin{array}{*{20}c} {{\rm x}_{\rm ij}^{\prime \prime } = \frac{{{\rm x}_{\rm ij}^{\prime } - {\rm x}_{\min } }}{{{\rm x}_{\max } - {\rm x}_{\min } }}} \\ {{\rm x}_{\min } = \min \{ {\rm x}_{\rm ij}^{\prime } \} } \\ {{\rm x}_{\max } = \max \{ {\rm x}_{\rm ij}^{\prime } \} } \\ \end{array} } \right..$$

## The DPC-GS-MND clustering algorithm

The key idea of density peaks clustering algorithm is based on the following two assumptions: (1) the cluster center is surrounded by data points not higher than its density; (2) the distance between the cluster center points and the higher density point is relatively far. The importance of density peaks clustering algorithm is the decision graph, that is, how to select the cluster centers more quickly and accurately^37^. This paper proposes an improved density peaks clustering algorithm called DPC-GS-MND, which is based on grid screening and mutual neighborhood degree. In the DPC-GS-MND algorithm, grid screening, mutual neighborhood degree and automatic center selection technologies are introduced to optimize and improve decision graphs drawing and cluster centers selection.

### Density peaks clustering

The density peaks clustering algorithm mainly includes three aspects: (1) Calculate local density ρi of each data point ${x}_{i}$, and minimum distance δi between ${x}_{i}$ and all other data points with higher density. (2) Obtain cluster centroids by the drawn decision graph according to ρi and δi. (3) Assign each remaining data point to the same cluster centroid as its nearest high-density neighbor.

#### Definition 1

Local Density. Given a dataset containing n data points *X* ($X=\left\{{x}_{1},{x}_{2},\dots ,{x}_{n}\right\}$), for $\forall {x}_{i},{x}_{j}\in X$, assuming that the local density of ${x}_{i}$ is ${\rho }_{i}$, then ${\rho }_{i}$ is given by Eq. (2);

2 $${\rho }_{i}= \sum_{{x}_{j}\in X}\chi \left(Eudist\left({x}_{i},{x}_{j}\right)-{dist}_{cutoff}\right),\quad \chi \left(x\right)=\left\{\begin{array}{ll}1,& x\le 0\\ 0,& x>0\end{array}\right.$$

where $Eudist\left({x}_{i},{x}_{j}\right)$ represents the Euclidean distance between data point ${x}_{i}$ and data point ${x}_{j}$, ${dist}_{cutoff}$ denotes a given cutoff distance.

#### Definition 2

High-density Distance. High-density distance is measured by computing the minimum distance between a data point and other higher-density data points. For the highest density data point, ${\delta }_{i}$ is calculated by Eq. (3), and for other data points, ${\delta }_{i}$ is computed by Eq. (4).

3 $${\delta }_{i}=\underset{{x}_{j}\in X,j\ne i}{\mathrm{max}}Eudist\left({x}_{i},{x}_{j}\right)$$

4 $${\delta }_{i}=\underset{j:{\rho }_{j}>{\rho }_{i}}{\mathrm{min}}Eudist\left({x}_{i},{x}_{j}\right)$$

#### Definition 3

Density Peak. The point whose distance is large and local density is large is defined as density peak. For $\forall {x}_{i}\in X$, the matrix $\left({\rho }_{i},{ \delta }_{i}\right)$ can be obtained by computing local density ${\rho }_{i}$ and distance from the high-density point ${\delta }_{i}$, and then the density peaks decision graph can be drawn. The density peak points have both high value of ${\rho }_{i}$ and ${\delta }_{i}$.

The detail process of DPC algorithm is shown as Algorithm 1.
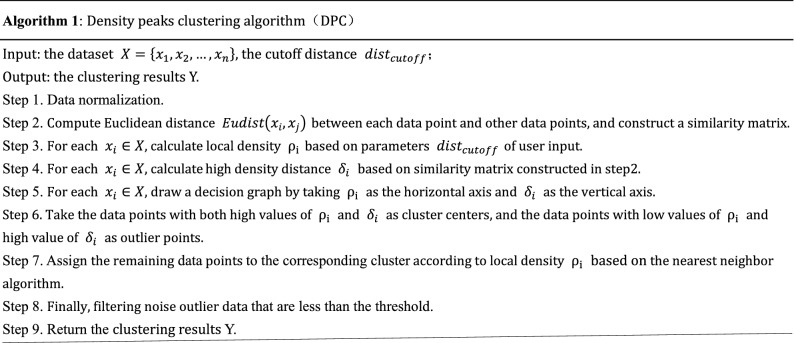


According to Algorithm 1, in Step 2, the space complexity increases significantly while calculating the distance matrix between all data points, and it limits the speed on large-scale datasets. In Step 3-Step 5, the definition of local density not considers the structural differences within the data, and it is difficult to obtain good clustering effect. In Step 6, the determination of cluster center requires human selection, which increases the uncertainty of clustering, especially when the human eye cannot accurately select the cluster center in some cases.

### Grid screening

DPC algorithm can efficiently detect anomalies and find clusters of arbitrary shapes. However, its space complexity is significantly increased when calculating the distance matrix between all data points, and it limits the speed of DPC application on large-scale datasets. The paper introduces the grid screening technology. First, it divides the whole data space by grid cells, and then maps the dataset to grid cells; Then it removes the sparse grid cells and focus on considering the data points in the rest dense grids. This greatly decreases memory requirements and time complexity.

#### **Definition 4 Grid Side Length**

Given a d-dimensional dataset containing n data points *X* ($X=\left\{{x}_{1},{x}_{2},\dots ,{x}_{n}\right\}$), for $\forall {x}_{i}\in X$, ${x}_{i}=\{{x}_{i}^{1},{x}_{i}^{2},\dots {x}_{i}^{dim},\dots ,{x}_{i}^{d}\}$, ${x}_{i}^{dim}$ denotes the dim-th feature of ${x}_{i}$, if all ${x}_{i}^{dim}\in \left[{l}_{i}\right.,\left.{h}_{i}\right)$(*dim* = 1,2,…,d), then $S=\left[{l}_{1}\right.,\left.{h}_{1}\right)*\left[{l}_{2}\right.,\left.{h}_{2}\right)*\dots *\left[{l}_{d}\right.,\left.{h}_{d}\right)$ represents a d-dimensional data space. Each dimension of the data space is divided into grid cells with equal sides and disjoint edges, and the grid side length $gsl$ is defined as follow.

5 $$gsl=\mu {\left(\prod_{i=1}^{d}\frac{{h}_{i}-{l}_{i}}{n}\right)}^\frac{1}{d}$$

where $\mu$ denotes the screening ratio, which is applied to adjust the size of the grid length $gsl$.

#### **Definition 5 Grid Cell Density**

Given a d-dimensional dataset containing n data points $X$ ($X=\left\{{x}_{1},{x}_{2},\dots ,{x}_{n}\right\}$), the data space is divided into grid cells $\left\{{u}_{1},{u}_{2},\dots ,{u}_{n}\right\}$ with grid side length $gsl$, and map X to the corresponding grid cells. The grid cell density of ${u}_{{\rm i}}$ is ${\rho }_{{u}_{i}}$ is defined as follow.

6 $${\rho }_{{u}_{i}}=count({G}_{{u}_{i}})$$

where $count({G}_{{u}_{i}})$ denotes the number of points in the cell with statistical grid number ${G}_{{u}_{i}}$.

### Mutual neighborhood degree

The local density defined in DPC algorithm does not consider the structural differences within the data. it is difficult to obtain good clustering effect. Therefore, the relative density and the neighbors of a sample can more accurately and effectively determine whether it is cluster center. Here, we compute the relative density in local area of the sample, rather than the relative density in the whole area.

#### Definition 6

KNN Local Density. Given a dataset containing n data points $X$ ($X=\left\{{x}_{1},{x}_{2},\dots ,{x}_{n}\right\}$), for $\forall {x}_{i}\in X$, $knn\left(i\right)$ represents the k nearest neighbors set of ${x}_{i}$ $and {x}_{j}\in knn\left(i\right)$. The KNN local density of ${\mathrm{x}}_{i}$ is ${\uprho }_{{\rm i}}$, which is defined as follow Eq. (7).

7 $${\rho }_{i}=\frac{\sum_{j=knn\left(i\right)}\sum_{v=knn\left(j\right)}Eu{dist\left({x}_{v},{x}_{j}\right)}^{2}}{2\cdot k\cdot \sum_{j=knn\left(i\right)}{Eudist\left({x}_{i},{x}_{j}\right)}^{2}}$$

where $Eudist\left({x}_{i},{x}_{j}\right)$ denotes the Euclidean distance between data point ${x}_{i}$ and data point ${x}_{j}$; k denotes neighbor points number.

#### Definition 7

Neighborhood Degree. Given a dataset containing n data points *X* ($X=\left\{{x}_{1},{x}_{2},\dots ,{x}_{n}\right\}$), for $\forall {x}_{i},{x}_{j}\in X$, the neighborhood degree is defined by distance between data points, and computation equation is as follow.

8 $$NDegree\left({x}_{i},{x}_{j}\right)=\left\{\begin{array}{ll}{e}^{- \frac{{Eudist\left({x}_{i},{ x}_{j}\right)}^{2}}{\frac{1}{N\dot k}\sum_{i=1}^{N}\sum_{{x}_{j}\in knn\left({x}_{i}\right)}{Eudist\left({x}_{i},{x}_{j}\right)}^{2}}} & {x}_{j}\in knn\left({x}_{i}\right)\\ 0& others\end{array}\right.$$

where $NDegree\left({x}_{i},{x}_{j}\right)$ represents the neighborhood degree between data point ${x}_{i}$ and data point ${x}_{j}$. The greater the distance between data point ${x}_{i}$ and data point ${x}_{j}$, the lower the similarity and the smaller the neighborhood degree.

#### Definition 8

Relative Neighborhood Degree. Given a dataset containing n data points *X* ($X=\left\{{x}_{1},{x}_{2},\dots ,{x}_{n}\right\}$), for $\forall {x}_{i},{x}_{j}\in X$, we introduce local neighborhood degree to compute the relative neighborhood degree of ${{\varvec{x}}}_{{\varvec{i}}}$ and ${{\varvec{x}}}_{{\varvec{j}}}$. The equation is as follow.

9 $$RNDegree\left({x}_{i},{x}_{j}\right)= \frac{1}{k+1}\sum_{{x}_{v}\in \left[knn\left({x}_{i}\right),{x}_{i}\right]}NDegree\left({x}_{v},{x}_{j}\right), i\ne j$$

where $knn\left({x}_{i}\right)$ denotes a set of the k nearest neighbors ${x}_{i}$, $NDegree\left({x}_{v},{x}_{j}\right)$ denotes the neighborhood degree of data point ${{\varvec{x}}}_{{\varvec{i}}}$ to data point ${{\varvec{x}}}_{{\varvec{j}}}$.

#### Definition 9

Mutual Neighborhood Degree. Given a dataset containing n data points *X* ($X=\left\{{x}_{1},{x}_{2},\dots ,{x}_{n}\right\}$), for ${\forall x}_{i},{x}_{j}\in X$, mutual neighborhood degree is defined based on relative neighborhood degree, as Eq. (10).

10 $$MNDegree\left({x}_{i},{x}_{j}\right)=RNDegree\left({x}_{i},{x}_{j}\right)\cdot RNDegree\left({x}_{j},{x}_{i}\right)$$

where $MNDegree\left({x}_{i},{x}_{j}\right)$ represents the mutual neighborhood degree of data point ${{\varvec{x}}}_{{\varvec{i}}}$ and data point ${{\varvec{x}}}_{{\varvec{j}}}$; $RNDegree\left({x}_{i},{x}_{j}\right)$ is the relative neighborhood degree of data point ${{\varvec{x}}}_{{\varvec{i}}}$ to data point ${{\varvec{x}}}_{{\varvec{j}}}$ and $RNDegree\left({x}_{j},{x}_{i}\right)$ is relative neighborhood degree of data point ${{\varvec{x}}}_{{\varvec{j}}}$ to data point ${{\varvec{x}}}_{{\varvec{i}}}$.

Here, the novel measure of mutual neighborhood degree between data points improves the density peaks clustering algorithm, and solves the problem that the density peaks clustering algorithm does not consider the structural differences within the data to find true local density.

### Automatic cluster center selection

In the DPC algorithm, the determination of cluster centers requires human selection, which leads to an increase in the uncertainty of clustering, especially in the case where the human eye cannot accurately select the cluster center in some cases. The choice of centers becomes very difficult. By comprehensively considering the two decision parameters ρ and δ of cluster center, a cluster center decision value is proposed:

11 $${\gamma }_{i}={P}_{i} * {\Delta }_{i}$$

where ${P}_{i}$ and ${\Delta }_{i}$ represent the normalized values of ${\uprho }_{{\rm i}}$ and ${\delta }_{i}$, respectively. The calculation equations as follow:

12 $${P}_{i}=\frac{{\rho }_{i}-{\rho }_{min}}{{\rho }_{max}-{\rho }_{min}}$$

13 $${\Delta }_{i}=\frac{{\delta }_{i}-{\delta }_{min}}{{\delta }_{max}-{\delta }_{min}}$$

where ${\rho }_{min}$ and ${\delta }_{min}$ represent the minimum value in ${\uprho }_{{\rm i}}$ and ${\delta }_{i}, {\rho }_{max}$ and ${\delta }_{max}$ are the maximum value in ${\uprho }_{{\rm i}}$ and ${\delta }_{i}$.

Obviously, the larger the $\gamma$ of the data point, the more likely it is the cluster center. We calculate the value of $\gamma$ and arrange ${\left\{{\gamma }_{i}\right\}}_{i=1}^{N}$ in descending order and plot on coordinate plane to get the density peaks decision graph. We can see that the γ value has obvious size boundaries. Therefore, the density peaks can be automatically selected by using a heuristic method to set a threshold. When the value of γ is greater than the threshold, the density peaks will be determined as the clustering centers.

### DPC-GS-MND: an improved DPC clustering algorithm

**Figure 3:**
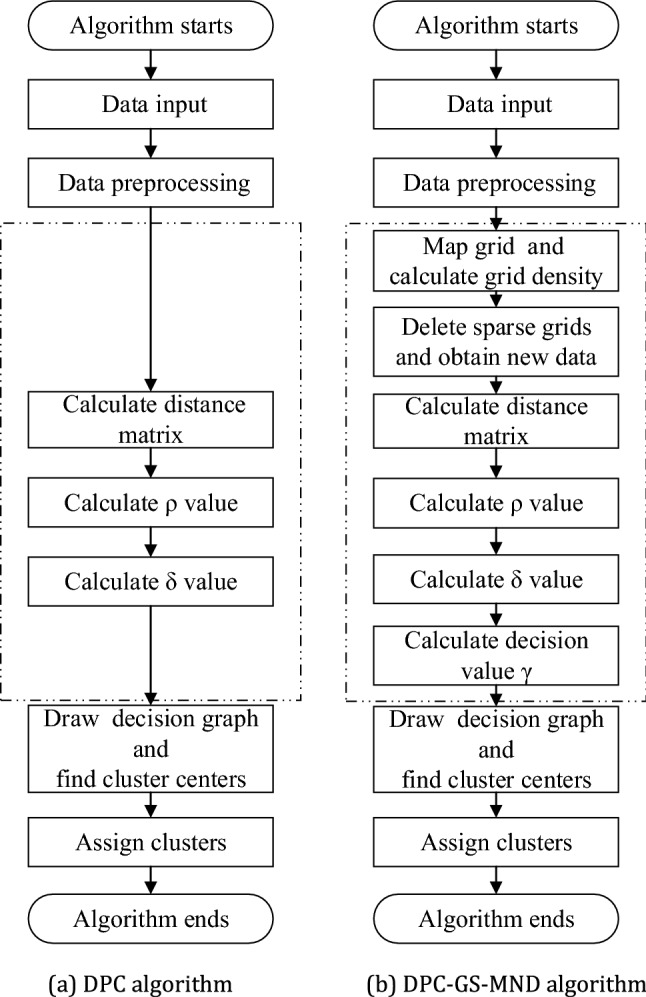
The algorithm flowchart.

First, DPC-GS-MND algorithm utilizes the idea of k-neighborhood to calculate the local density of data points and find the density peaks, and then assigns the k nearest neighbors to their corresponding clusters. Secondly, it computes the mutual neighborhood degree between data points, and then finds the closest unallocated data points according to the mutual neighborhood degree, next, assigns them to the relative clusters. Finally, it repeats this operation until all data points are allocated. The DPC-GS-MND algorithm flowchart is shown in the follow Fig. 3.

The detailed process of the proposed DPC-GS-MND algorithm is as follow Algorithm 2.
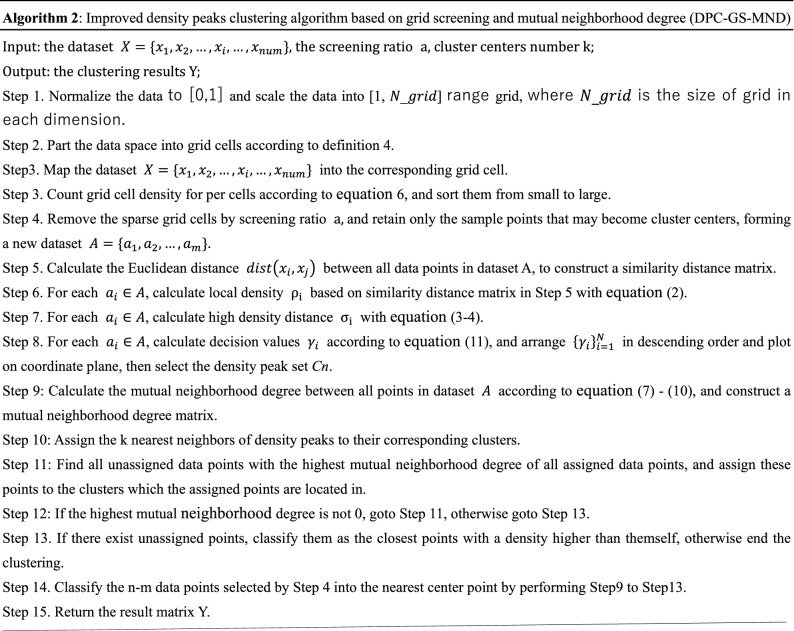


For a dataset $X$ with data size n, $X=\left\{{x}_{1},{x}_{2},\dots ,{x}_{n}\right\}$, the DPC-GS-MND algorithm only needs cluster the new dataset $A=\{{a}_{1},{a}_{2},\dots ,{a}_{m}\}, {a}_{i}\in X$ consisting of $m\left(m\ll n\right)$ elements contained in the dense grids. The complexity of DPC-GS-MND algorithm is much smaller than that of DPC algorithm, especially when the value of n is extremely large. The DPC-GS-MND algorithm effectively solves the problem of poor clustering of data with varying density degree and the associated allocation errors. Regardless of the running time or the required memory, its execution efficiency is much higher than the DPC algorithm.

## Evaluation

Finally, we verify our network anomaly detection methods through the various experiments on the real dataset and compare the results. The following experiments are to evaluate the benefits of the effectiveness of the DPC-GS-MND method for detecting various network anomaly traffic.

### Experimental setup

The platform used for all these experiments integrates an Intel Xeon E5-2630 @ 2.3 GHz, 64 GB RAM. The algorithm is implemented with Python 3.2, Network X 1.9.1 and Sklearn 0.2.0 on Ubuntu 17 64 OS are implemented as software frameworks.

We utilize accuracy rate to evaluate the experiment results in this paper, which is given by the follow Eq. (14).

14 $$Accuracy Rate=\frac{{\sum }_{i=1}^{m}({TP}_{i}+{TN}_{i})}{{\sum }_{i=1}^{m}({TP}_{i}+{TN}_{i}+{FP}_{i}+{FN}_{i})} ,$$

where *m* is the number of network anomaly types, ${n}_{ij}$ denotes the number of *i-*type network anomalies clustered to be *j* type. ${\mathrm{TP}}_{{\rm i}}$, ${\mathrm{FP}}_{{\rm i}}$, ${\mathrm{FN}}_{{\rm i}}$ and ${\mathrm{TN}}_{{\rm i}}$ are defined as: ${\mathrm{TP}}_{{\rm i}}={\mathrm{n}}_{{\rm ii}}$, ${\mathrm{FP}}_{{\rm i}}=\sum_{{\rm j}\ne \mathrm{i}}{\mathrm{n}}_{{\rm ji}}$, ${\mathrm{FN}}_{{\rm i}}=\sum_{{\rm i}\ne \mathrm{j}}{\mathrm{n}}_{{\rm ij}}$ and ${\mathrm{TN}}_{{\rm i}}=\sum_{{\rm j}\ne \mathrm{i}}{\mathrm{n}}_{{\rm jj}}$.

### Experimental results

We have developed and studied the network anomaly detection model based on DPC-GS-MND clustering algorithm that uses only observable aspects of network traffic.

#### **Experiments 1**

**Figure 4:**
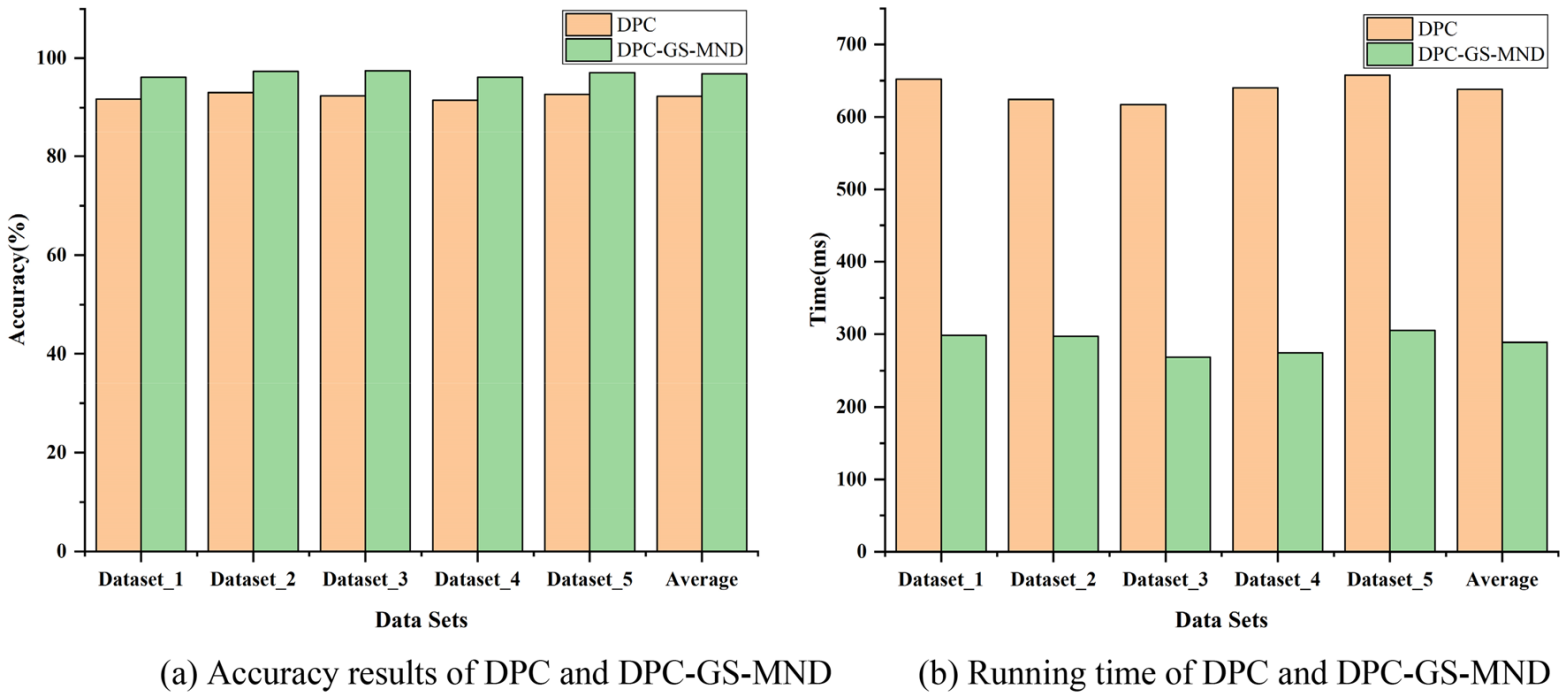
Comparison Accuracy and Time of DPC and DPC-GS-MND.

We performed experiments on five sub-datasets to evaluate effectiveness of the proposed network traffic anomaly detection algorithm DPC-GS-MND. In order to verify the overall performance, each sub-dataset is composed of randomly selected 5000 DoS attack samples and 5000 normal samples in 10% of KDDCUP99, as well as other three attack type samples. The experiments were performed on both basic DPC algorithm and DPC-GS-MND algorithm. Each experiment was repeated 5 times, and the calculation parameters were averaged. Figure 4 shows the comparison results of the two in clustering accuracy and clustering time.

Figure 4 shows that each time a different dataset is selected, the proposed DPC-GS-MND algorithm has different detection accuracy and performance, but the fluctuations are within the acceptable range. The reason is that the algorithm has ability to recognize different anomalies. By comparing the running accuracy of different datasets, it is verified that the DPC-GS-MND algorithm is superior to the anomaly detection accuracy and the detection effect is better than DPC algorithm. The DPC-GS-MND algorithm can quickly and accurately identify anomalies.

#### **Experiments 2**

**Figure 5:**
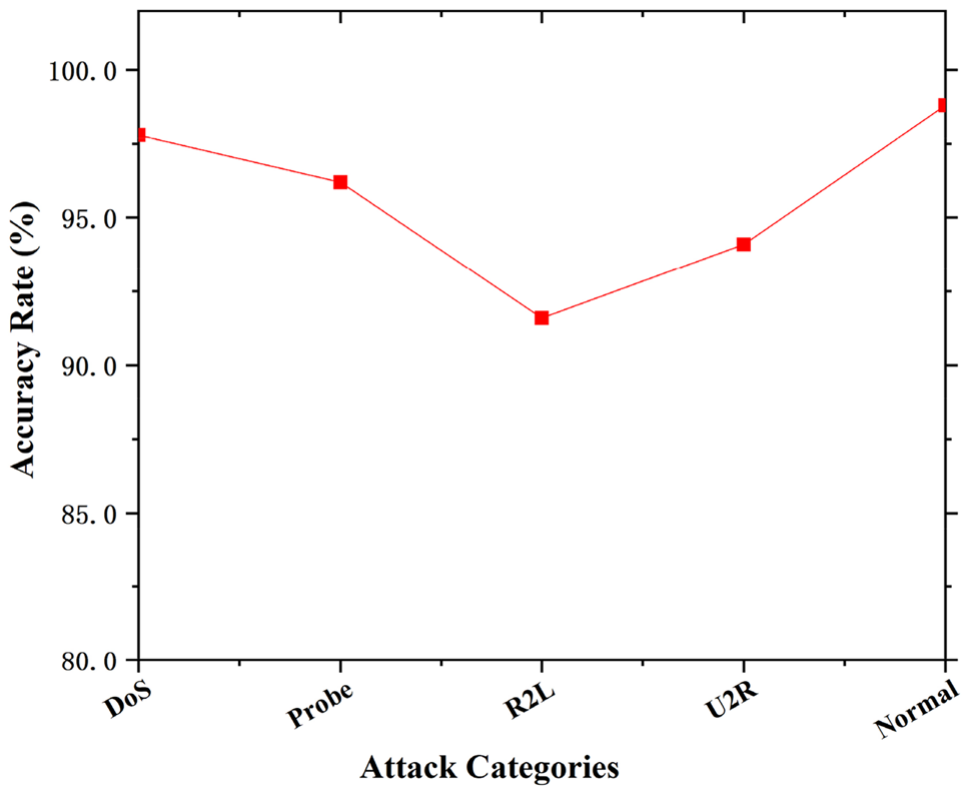
Detection accuracies on single attack type data.

 We conducted experiments on five single types to verify the detection effect on a single attack type. The result is shown in Fig. 5. Since the feature value of the R2L attack pattern is changeable, it is much similar to normal data and not easy to be detected. Therefore, in addition to R2L, the other three types of attack are relatively good, each detection accuracy exceeds 93%.

#### **Experiments 3**

 In this paper, we introduce grid screening and mutual neighborhood degree technology to improve the DPC clustering algorithm. In these experiments, we need to confirm the effectiveness of grid screening technology and mutual neighborhood degree technology. We compare the network anomaly detection accuracies and running time of DPC, DPC-GS (improved DPC with GS), DPC-MND (improved DPC with MND) and DPC-GS-MND algorithms.

**Figure 6:**
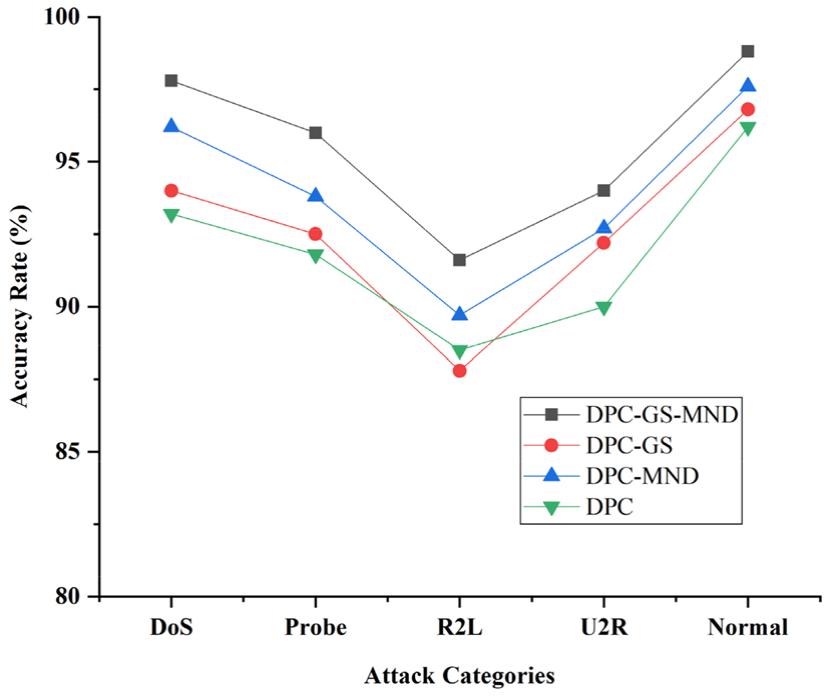
Anomaly detection accuracy comparing of DPC-GS-MND, DPC-GS, DPC-GS and DPC.

**Figure 7:**
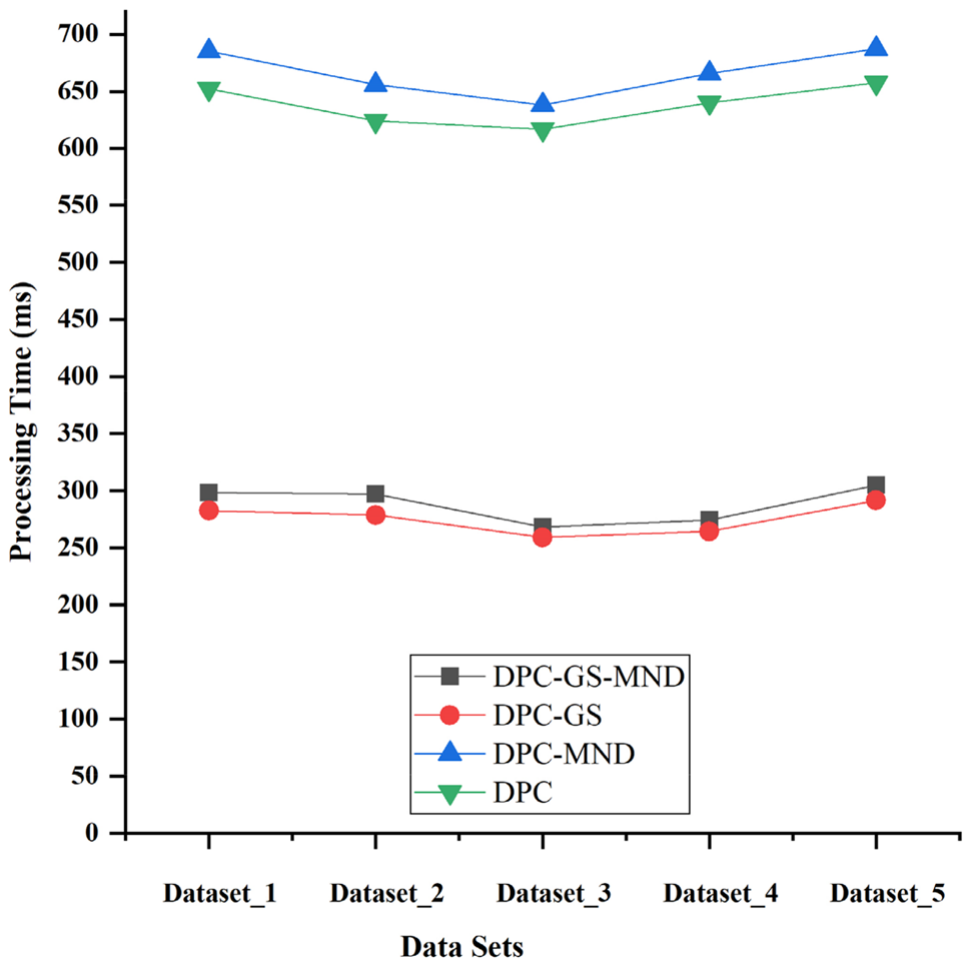
Anomaly detection running time comparing of DPC-GS-MND, DPC-GS, DPC-GS and DPC.

As shown in Figs. 6 and 7, the experimental results show that, compared with DPC-GS, DPC-MND and DPC, the DPC-GS-MND algorithm has much higher anomaly detection accuracy. The DPC-GS-MND algorithm has much lower running time than DPC-MND and DPC. DPC-MND has better anomaly detection accuracy than DPC-GS, but DPC-GS has a shorter running time. This shows that the introduced grid screening technology can improve the computational performance, and the introduced mutual neighborhood degree technology can effectively improve the detection accuracy.

#### **Experiments 4**

In order to confirm the improvement of the proposed DPC-GS-MND algorithm, we compared the network anomaly detection accuracy with different algorithms on a same dataset, including DPCG (2016) [2033], MDPCA (2019) [2034], DPC-DLP (2019) [2031] and DPC-GS-MND algorithm.

The anomaly detection accuracy rate and running time of the four algorithms are shown in Table 3. Among all the four clustering algorithms, network anomaly detection using the DPC-GS-MND algorithm can provide the best accuracy rate and relatively short running time. This shows that the DPC-GS-MND algorithm has better anomaly detection accuracy than MDPCA, DPC-DLP and DPCG. The DPC-GS-MND algorithm takes less time than MDPCA and DPC-DLP, but takes a little more time than DPCG. The accuracy of DPCG is lower than that of MDPCA and DPC-DLP, but the running time is shorter. This is because both DPC-GS-MND and DPCG utilize grids to greatly improve the running efficiency of algorithm, while DPC-GS-MND takes some time to further compute the mutual neighborhood degree.

**Table 3:** Accuracy rate and running time of four algorithms.

Detection method	Accuracy rate (%)	Running time (ms)
MDPCA	90.57	378.2
DPCG	94.25	274.8
DPC-DLP	95.96	452.7
DPC-GS-MND	96.83	288.6

#### **Experiments 5**

 In order to further confirm the availability of the DPC-GS-MND algorithm for network anomaly detection, we also have some experiments on CIC-IDS-2017, which is a latest network traffic dataset and covers 11 common attacks including Bot, DoS, DDoS, SQL Injection, Brute Force, Infiltration, Port scan and XSS.

**Figure 8:**
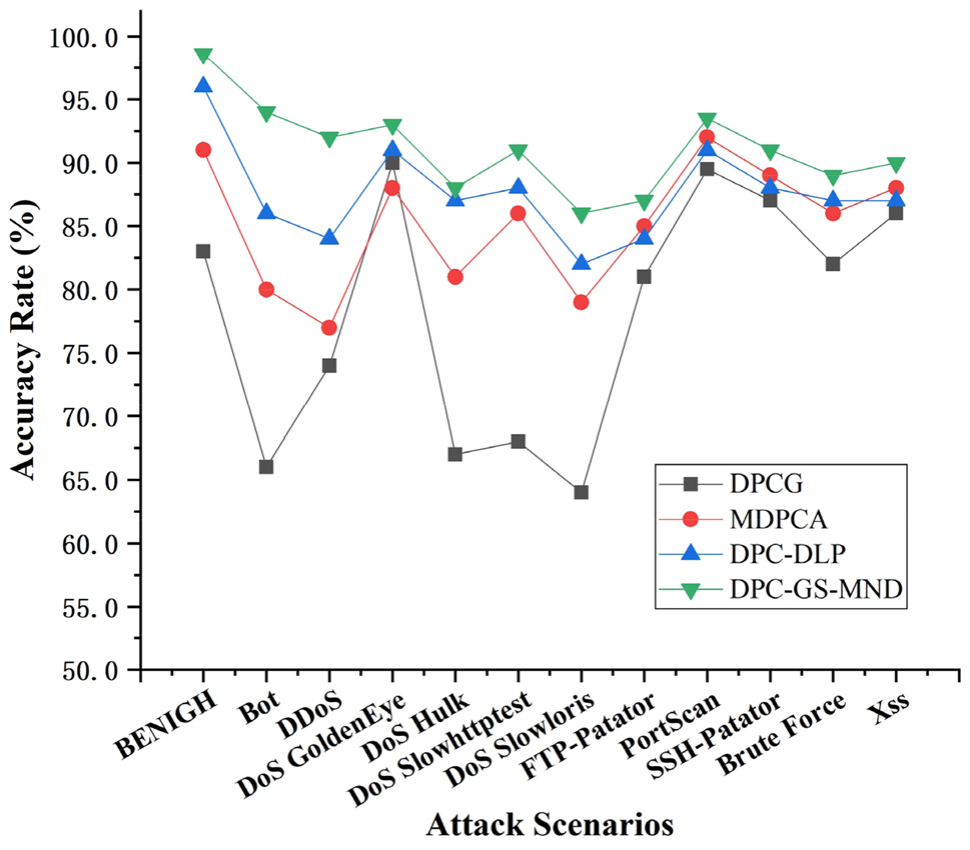
Anomaly detection accuracies on CIC-IDS-2017.

As shown in Fig. 8, the experimental results show that the DPC-GS-MND algorithm has a higher anomaly detection accuracy than DPCG, MDPCA and DPC-DLP on the dataset CIC-IDS-2017. Based on the Experiments 4 to Experiments 6, the DPC-GS-MND algorithm has better result both on real-world datasets KDDCUP99 and CIC-IDS-2017.

Through our experimental evaluation, we clearly proved that the network traffic anomaly detection model based on the DPC-GS-MND algorithm outperforms DPC, DPCG, MDPCA and DPC-DLP. The proposed DPC-GS-MND algorithm improves both the accuracy and efficiency of network traffic anomaly detection.

## Conclusion and future work

In this paper, we propose and evaluate a novel improved network traffic anomaly detection method named DPC-GS-MND. In order to achieve efficient and accurate detection of abnormal traffic, an improved density peaks clustering algorithm based on grid screening and mutual neighborhood degree is proposed. Experiment results on well-known datasets show that the proposed DPC-GS-MND method was able to identify anomaly with higher detection accuracy and less running time.

In future research, we hope to extend the work to the following two major directions. (1) Adaptive k value selection: The DPC-GS-MND algorithm uses k nearest neighbors, the value of parameter k still needs to be manually determined and the adaptive determination of k value will be the next work. (2) Data reduction: data reduction includes network traffic sampling and important features extraction. We need to further study malware traffic sampling and its representative features extraction to improve the efficiency and accuracy of anomaly detection. During the process of network traffic sampling, more attention should be paid to the unbalanced features of network traffic data.
